# An Unlikely Cause of Groin Pain: Obturator Externus Tear in a Professional Soccer Player

**DOI:** 10.7759/cureus.44612

**Published:** 2023-09-03

**Authors:** Rui Silva, Adriana Pereira, Sérgio Rodrigues-Gomes, Tiago Lopes

**Affiliations:** 1 Sports Medicine, Futebol Clube do Porto, Porto, PRT; 2 Physical Medicine and Rehabilitation, Centro de Medicina de Reabilitação de Alcoitão, Cascais, PRT; 3 Radiology, Espregueira-Mendes Sports Center, Porto, PRT; 4 Radiology, Grupo Unilabs, Porto, PRT; 5 Physical Medicine and Rehabilitation, Centro de Reabilitação do Norte, Porto, PRT; 6 Physical Medicine and Rehabilitation, Espregueira-Mendes Sports Center, Porto, PRT

**Keywords:** muscle injuries, return to play, mri, groin pain, soccer player, obturator externus tear

## Abstract

Groin pain is a common and complex problem in athletes, especially soccer players, associated with a wide variety of possible injuries in numerous anatomical structures. One of the causes of groin pain is damage to the deep muscles of the hip region, with isolated traumatic injury of the obturator externus muscle rarely described and probably underdiagnosed. This report describes a clinical case of a soccer player who presented with acute hip pain and buttock pain resulting from a rapid change of position in load, associated with pain with active hip external rotation and passive internal rotation. MRI demonstrated the presence of subaponeurotic/myo-aponeurotic obturator externus muscle tear. A conservative treatment was decided, targeting pain reduction and progressing range of motion gain and muscle strengthening of the stabilizing muscles of the pelvis and hip, and subsequently, it led to re-athletisation, with soccer-specific exercises. Return to play was 23 days after injury. This case shows that a high level of suspicion is necessary for the correct diagnosis; treatment is generally conservative and the isolated rupture of the external obturator can be considered relatively benign. However, it has the potential to be associated with a long period of absence from training and games.

## Introduction

Groin pain in athletes, especially in soccer players, is a common problem (5-28% annual incidence and 12-16% of all injuries in football) and known for being a complex issue. Long periods of training and game absence due to groin pain are not uncommon (more than 50% lead to absence of more than one week) [1]. The wide variety of possible injuries in numerous anatomical structures and the high prevalence of “abnormal findings” in asymptomatic athletes contribute to the complexity. The nomenclature associated with groin pain is also heterogeneous, which also contributes to the difficulty in defining the various clinical entities. Therefore, a multidisciplinary approach is recommended [1,2].

One of the causes of groin pain is damage to the deep muscles of the hip region, including the short lateral rotators tear (piriformis, obturator externus, superior and inferior gemelli, obturador internus, and quadratus femoris) [1-4]. Nevertheless, isolated traumatic injury of the obturator externus muscle is rarely described with only a few case reports being described in the literature [3,5]. So far the case reports that described the injury raise the suspicion that there is a correlation with ball-kicking sports (after long-distance shooting or passing), such as soccer, rugby, and American football [3,6].

The obturator externus originates from the external bony margin of the obturator foramen with a cylindrical tendon passing under the femoral neck and inserting in the trochanteric fossa [7,8], 25-38 mm distal to the piriformis fossa, with a completely different trajectory from the other external rotators [9]. The primary function of the obturator externus is to externally rotate the hip when in neutral position and ﬂexed at 90 degrees, while the secondary action is to adduct the hip when in ﬂexion and stabilize it [5-7,10].

There is no established mechanism of injury, but there are several described: an unstable position of the pelvis associated with an increase or change of the load on the affected hip, a repetitive loading of the extremity while kicking the ball with the affected or unaffected side, unstable change of direction trying to control the ball, anterior or lateral hip slide in an unstable position, repetitive ball kicking, and kicking the ball in an unstable position [3-5]. The musculoskeletal physical examination to differentiate obturator externus injury from other potentially more common injuries is also unspecific, so a high index of clinical suspicion is required [3,6,8]. However, most patients present anterior hip pain. Clinical examination elicits pain associated with hip flexion and passive internal or external rotation against resistance, sometimes suggestive of intraarticular joint pathology [3,5,6,10].

Due to the deep location of the muscle, ultrasound cannot reliably identify its injury. Thus, whenever there is suspicion of injury to the deep hip muscles, an MRI has to be performed to identify the location and degree of the injury [3-6,8]. Correctly recognizing obturator externus injury allows for conservative management (physical therapy and non-steroidal or analgesic medication) and a relatively short recovery period, which can vary from 10 to 21 days [3,6,8].

In this report, we present the description of case of an elite professional soccer player who suffered an obturator externus tear during a game and the progression until his return to play (RTP).

## Case presentation

**Figure 1:**
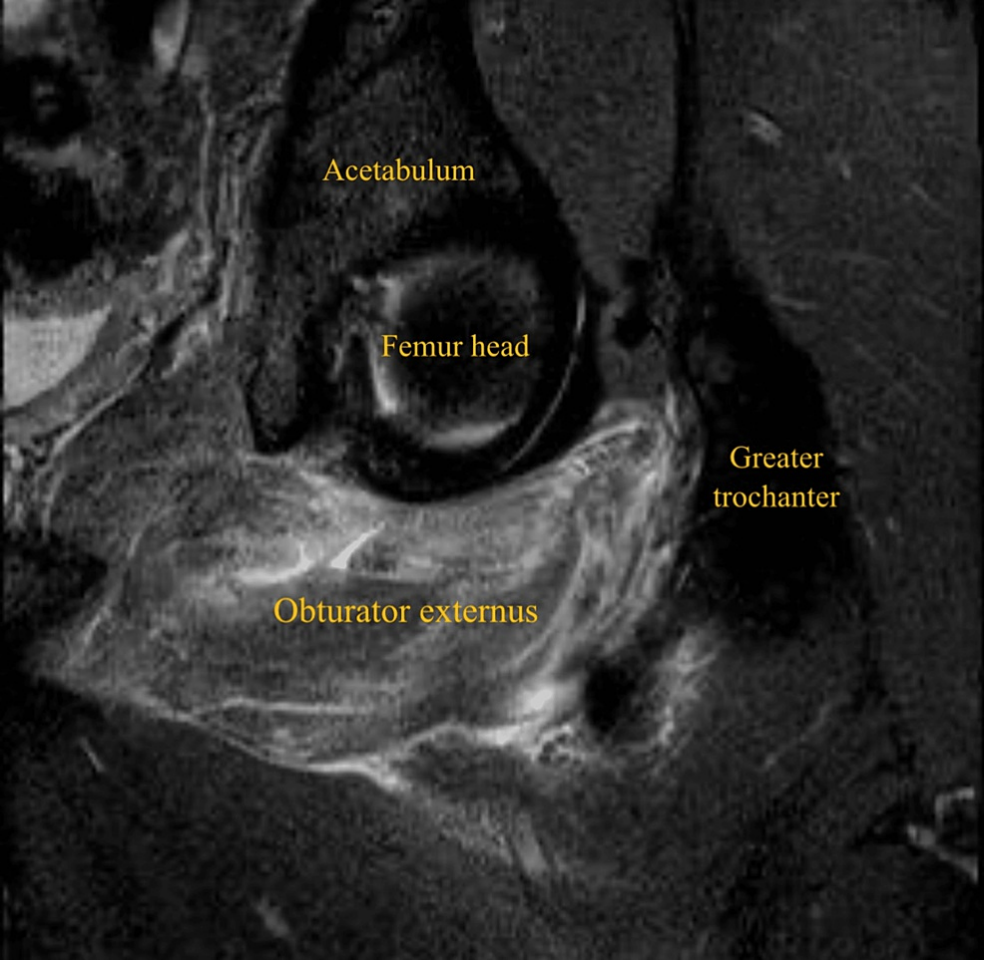
Coronal short-tau inversion recovery (STIR) MRI of the left thigh showing left obturator externus tear

**Figure 2:**
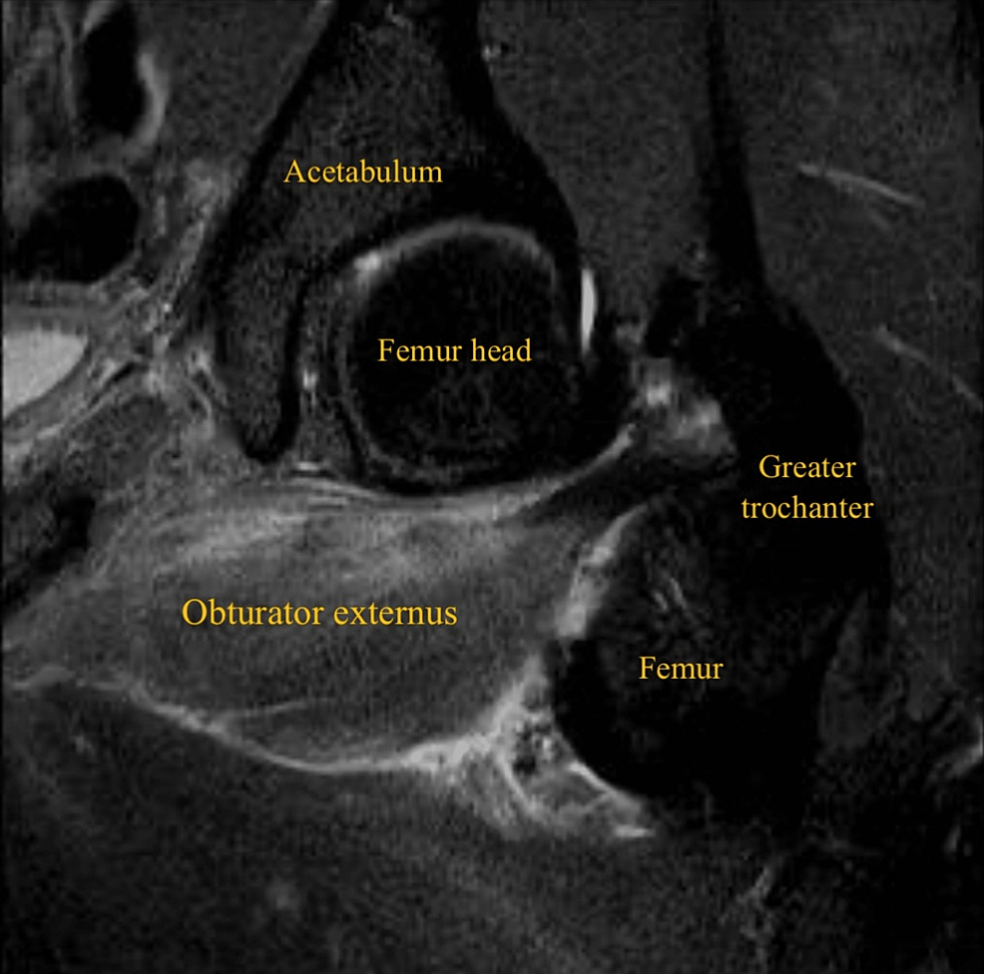
Coronal oblique short-tau inversion recovery (STIR) MRI of the left thigh showing left obturator externus tear

**Figure 3:**
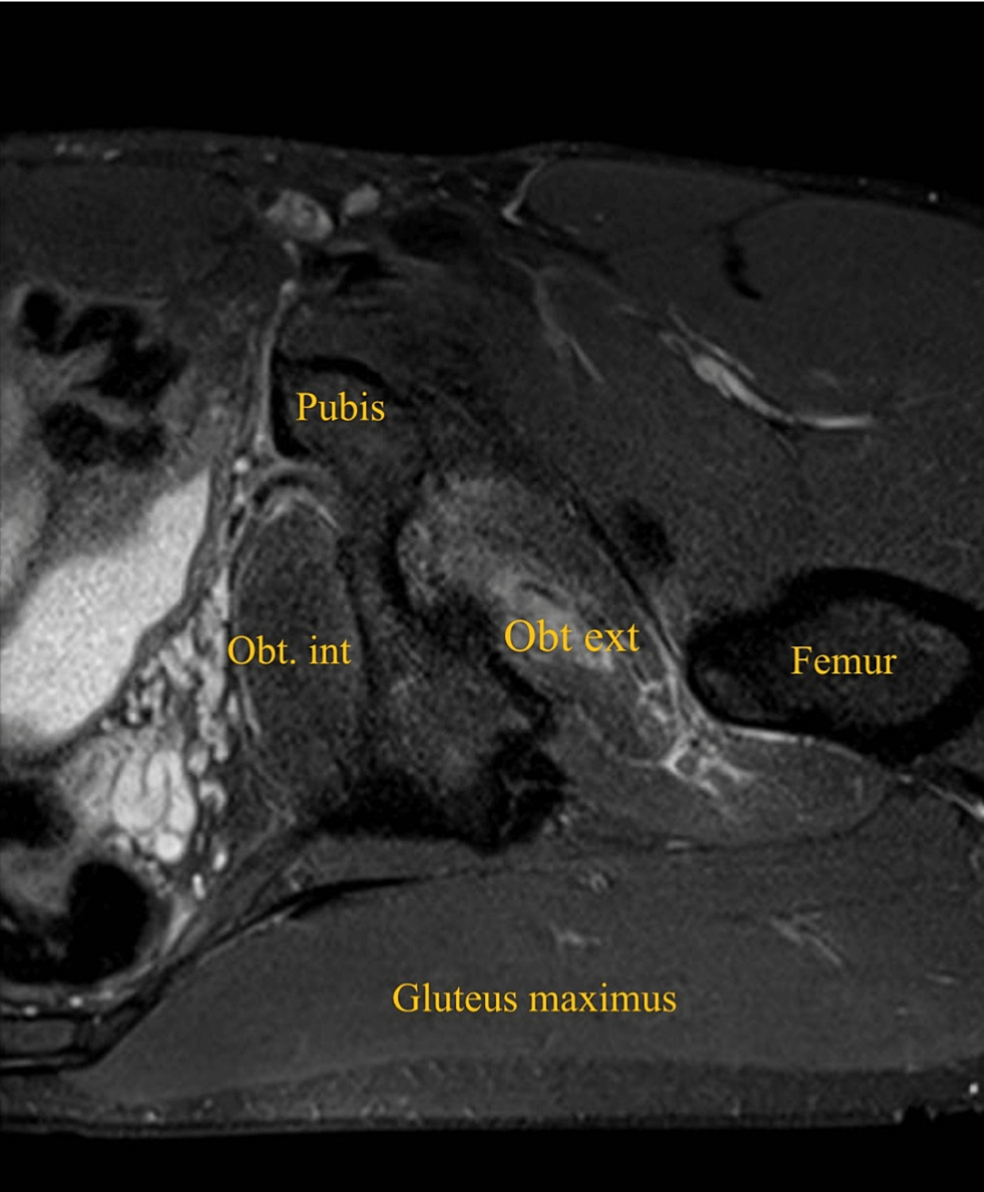
Axial short-tau inversion recovery (STIR) MRI of the left thigh showing left obturator externus tear Obt. ext: obturator externus; Obt. int: obturator internus

**Figure 4:**
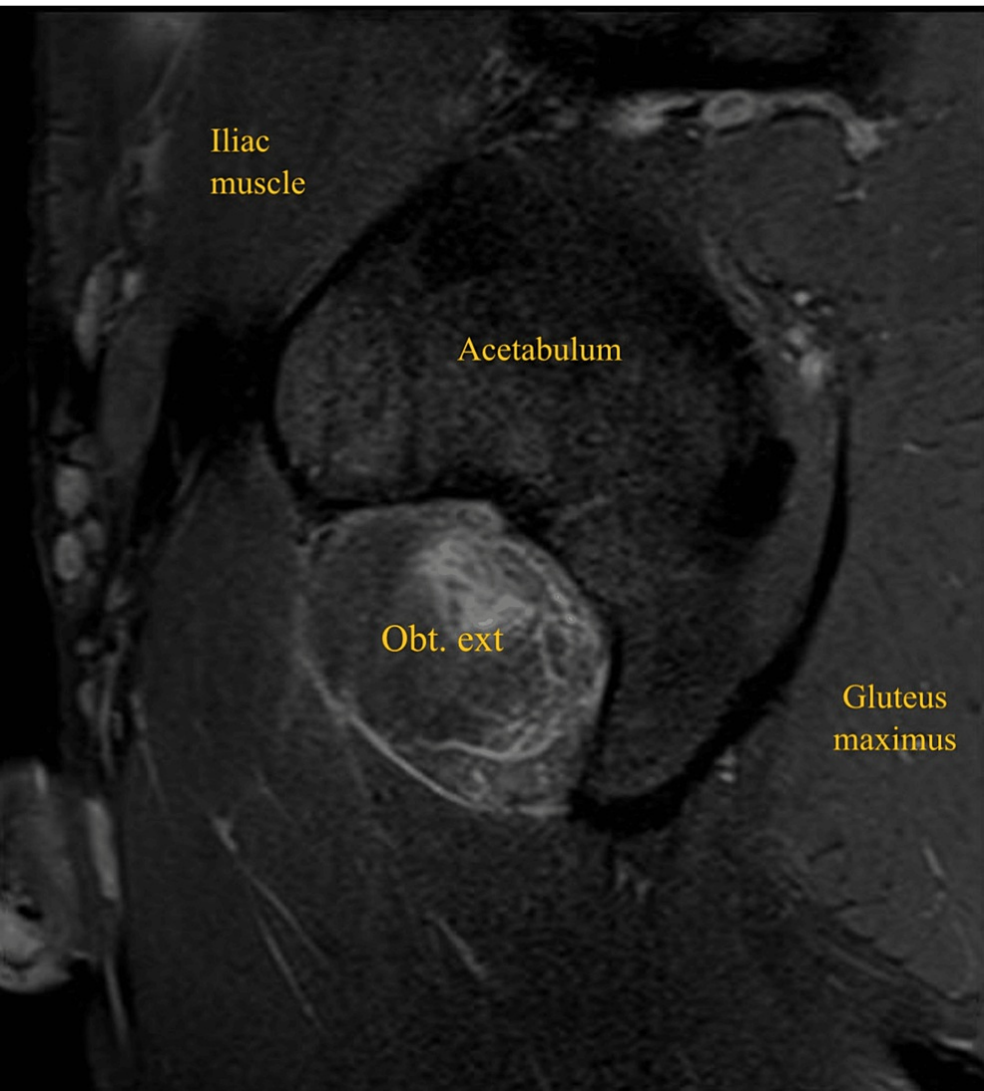
Sagittal short-tau inversion recovery (STIR) MRI of the left thigh showing left obturator externus tear Obt. ext: obturator externus

A 32-year-old male patient, professional soccer player, presented with acute left groin pain and buttock pain during an in-season training session, with a visual analog scale (VAS) score of 8/10, resulting from a rapid and loaded hip intra-rotation movement, with the hip and knee flexed at approximately 90 degrees, in a fast change direction and cutting movement to the attack, with immediate functional incapacity. He denied any numbness, weakness, or radiating pain to the leg. On physical examination, there was no bruising, ecchymosis, edema, or swelling. There was slight pain and tenderness on palpation of the ischial tuberosity. No abnormalities were found in the specific testing of the adductor, hamstring, or iliopsoas muscles. Active hip external rotation and passive hip internal rotation at 90 degrees of knee and hip elicited pain. The player was able to walk without limping. X-ray of the left hip was done to exclude hip joint pathology, including femoroacetabular impingement. MRI revealed a posterior subaponeurotic/myo-aponeurotic tear of the left obturator externus muscle, extending from its pubic origin to the lateral myotendinous junction, measuring 10 cm in the transverse plane and 3.5 cm in the anteroposterior diameter, associated with quadratus femoris and obturator internus muscles interstitial edema (Figures 1-4).

The patient followed a rehabilitation protocol (Table 1), with the transition criteria between phases being strictly clinical. We started with relative rest, control of pain, and application of electrotherapy, magnetotherapy, and cryotherapy. After that, the main objective was the restoration of range of motion (ROM), and strengthening exercises of the pelvis and hip stabilizing muscles (starting with isometric and gradually increasing to progressive resistance, as tolerated) were also included. During the rehabilitation period, the patient performed core exercises, upper and lower body strengthening, and cardiovascular conditioning. Finally, he started a re-athletisation period, with soccer-specific exercises, until he was ready to RTP. He returned to professional competition 23 days after injury. One year after the injury, no reinjury occurred and the athlete is fully satisfied, with absolutely no implications on his physical performance.

**Table 1:** Rehabilitation program TENS: transcutaneous electrical nerve stimulation; ROM: range of motion; PNF: proprioceptive neuromuscular facilitation; GPS: global positioning system

Phases	Rehabilitation Program	Rehabilitation Goals	Progressive Criteria
Phase I (0-5 days)	Static cryotherapy 10 minutes; Magnetotherapy 3-6mT, 6Hz, 15 minutes; TENS 100Hz, 15 minutes; Gentle massage; Active-assisted mobilization of the hip; *Local and remote myofascial technique.	Control of the inflammatory process; Control of pain; Gain in mobility.	Ability to walk without pain; Absence of pain with maximal isometric contraction; Absence of pain with passive/active stretching.
	Gymnasium: Isometric exercises with manual resistance for flexion, extension, adduction, abduction, external and internal rotation of the hip; Core exercises: isometric front plank and gluteal plank.		
Phase II (6-13 days)	Magnetotherapy 5-10mT, 10Hz, 15 minutes; Techniques for active ROM in the hip, pelvic girdle and lower back; Local and distant myofascial technique; Deep massage with the release of trigger points; Hydrokinesiotherapy.	Gain in mobility; Improvement of strength; Improved neuromuscular coordination; Global reconditioning of the effort; Integration of the athletic gesture, without the ball.	Complete ROM; Absence of pain during maximal concentric and submaximal eccentric isotonic contractions; Motor standard normalization; Slow running on treadmill and bicycle without pain.
	Gymnasium: Progression to concentric and later eccentric dynamic exercises with increasing external loading that include flexion, extension, adduction, abduction, external and internal rotation of the hip; Core exercises: static planks (high plank, side plank, reverse plank) dynamic planks with increasing instability (reverse plank leg raise, reverse plank hip raise, side plank hip raise); PNF diagonals without and with progressive elastic resistance; Proprioceptive training; Bicycle and Treadmill; Technical gestures without ball.		
Phase III (14-23 days)	Techniques to improve the mobility of the lumbar spine, pelvis and hip; Local and distant myofascial technique; Deep massage with the release of trigger points.	Muscle and neuromuscular potentiation; Normalization of technical gesture; Reconditioning to effort; Prevent relapse.	Return to play Criteria: Absence of pain in all technical skills; Absence of pain when changing direction and at maximum speeds; GPS with 95-100% of pre-injury values; Carry out three complete soccer pratices, without limitations.
	Gymnasium: No restriction on the type of strengthening exercises (e.g. jumping jacks, Swiss ball plank, glute bridge, normal deadlift, sumo deadlift, bilateral and unilateral Romanian deadlift, squat, Bulgarian split squat, box squat); Plyometric training.		
	Soccer field: Technical skills with ball; Running, with progression of distance, speed, and changes of direction; Starts in 16 day soccer pratices without contact; Performs normal and full training in 20 day; Game in 23day.		

## Discussion

Obturator externus tear is extremely rare, with very little description in the literature [3,5], which makes this case rvery interesting, but also a great challenge to discuss. There is controversy regarding clinical, imaging, optimal treatment, and prognosis. 

Indeed, the most frequently described causes of groin pain are adductor-, iliopsoas-, and inguinal-related groin pain [2] However, injury to the external rotator muscles is also a cause of groin pain and should therefore be taken into account [1,6]. Several mechanisms of injury are described, but none very specific, and the most commonly described involve the movements that our athlete performed: rapid and unstable change of speed and load, associated with flexion and internal rotation. Despite the various mechanisms described, it is proposed that these injuries occur due to the attempt to stabilize the hip during a demanding activity that involves a combination of forces acting on the hip [3].

In most cases, athletes with external obturator injury present pain in the anterior region of the hip, and very rarely pain in the buttock, which may be caused by many other pathologies, most of them more common than injury to the obturator externus [3]. In fact, this injury is often misdiagnosed as a hip adductor muscle injury due to the location of the pain, which may be related to the common innervation of these muscles by the obturator nerve [5]. It is proposed that the most suggestive clinical condition is pain in the anterior hip, acute or subacute, triggered by an unstable situation in high-demand sports, associated with pain during passive hip rotation or against resistance with the hip in 90º flexion [3]. Another important feature pain is not elicited with palpation because of the deep location of the muscle the external obturator [5]. Despite these characteristics, the clinical diagnosis remains a challenge, and ultrsound typically does not have sufficient resolution in depth for the correct diagnosis. Therefore, MRI is the gold standard, allowing assessment of the location and severity of the lesion [5,6]. The clinical presentation and physical examination are very unspecific, so the diagnosis is essentially a diagnosis of exclusion of the most common causes. It requires a strong clinical suspicion as well as an imaging diagnosis by MRI, as in our athlete.

There is no consensus between rehabilitation protocols. Rehabilitation programs typically last two to three weeks, starting with relative rest and control of pain complaints and progressing to recovery of ROM and muscle strengthening, from isometric to dynamic, according to tolerance. Subsequently, the training of exercises specific to the modality begins until the athlete is ready to RTP. Core strengthening and aerobic training are also important throughout this process [3]. In general, this was the established rehabilitation protocol in this case report, with a resolution of the clinical picture.

The prognosis for external obturator injury is generally benign, with faster resolution than injury to other hip muscles. Although it varies, the average RTP time varies between 18.2 ± 3 days and 11.5 ± 8,8 days [3]. This clinical case is in agreement, with a return to competition after 23 days.

## Conclusions

Obturator externus tear is a rare injury and can be the cause of groin pain in a soccer players. A high level of suspicion and a correct diagnosis of external obturator tear with the use of MRI allows targeted conservative treatment and a shorter recovery time. The treatment of this injury is conservative, so we can consider obturator externus tear as a benign lesion. Nevertheless, it has the potential to be associated with a long period of absence from training and games.

## References

[1] Werner J, Hägglund M, Waldén M, Ekstrand J (2009). UEFA injury study: a prospective study of hip and groin injuries in professional football over seven consecutive seasons. Br J Sports Med.

[2] Weir A, Brukner P, Delahunt E (2015). Doha agreement meeting on terminology and definitions in groin pain in athletes. Br J Sports Med.

[3] Wong-On M, Turmo-Garuz A, Arriaza R (2018). Injuries of the obturator muscles in professional soccer players. Knee Surg Sports Traumatol Arthrosc.

[4] Toliopoulos A (2022). Isolated obturator internus muscle strain injury in a professional football player: a case report. Cureus.

[5] Valente HG, Marques FO, De Souza LS, Abib RT, Ribeiro DC (2011). Injury of the external obturator muscle in professional soccer athletes. Rev Bras Med Esporte.

[6] Rhim HC, Gureck AE, Jang KM (2022). Acute obturator externus injury in professional soccer players: a case series. Medicina (Kaunas).

[7] Gudena R, Alzahrani A, Railton P, Powell J, Ganz R (2015). The anatomy and function of the obturator externus. Hip Int.

[8] Khodaee M, Jones D, Spittler J (2015). Obturator internus and obturator externus strain in a high school quarterback. Asian J Sports Med.

[9] Kawaguchi Y, Otani T, Fujii H, Hayama T, Marumo K, Saito M (2021). Functional and clinical anatomy of the obturator externus muscle: cadaveric studies and clinical findings for total hip arthroplasty in the posterior approach. J Orthop.

[10] Kim SH, Kim DH, Yoon DM, Yoon KB (2015). Clinical effectiveness of the obturator externus muscle injection in chronic pelvic pain patients. Pain Pract.

